# Application of Zinc Oxide Nanoparticles to Mitigate Cadmium Toxicity: Mechanisms and Future Prospects

**DOI:** 10.3390/plants13121706

**Published:** 2024-06-19

**Authors:** Muhammad Umair Hassan, Guoqin Huang, Fasih Ullah Haider, Tahir Abbas Khan, Mehmood Ali Noor, Fang Luo, Quan Zhou, Binjuan Yang, Muhammad Inzamam Ul Haq, Muhammad Mahmood Iqbal

**Affiliations:** 1 Research Center on Ecological Sciences, Jiangxi Agricultural University, Nanchang 330045, China; muhassanuaf@gmail.com (M.U.H.); tahirsargani@gmail.com (T.A.K.); mehmood2017@gmail.com (M.A.N.); jxndlf920@126.com (F.L.); zhouquanyilang@163.com (Q.Z.); yangbinjuan@jxau.edu.cn (B.Y.); 2 South China Botanical Garden, Guangzhou 510520, China; haider281@scbg.ac.cn; 3 Department of Agronomy, University of Agriculture Faisalabad, Faisalabad 38040, Pakistan; inzamamh79@gmail.com; 4 Agronomy (Forage Production) Section, Ayub Agricultural Research Institute, Faisalabad 38040, Pakistan; mahmkhokhar@gmail.com

**Keywords:** cadmium, growth, photosynthesis, osmolyte, zinc nanoparticles

## Abstract

Cadmium (Cd), as the most prevalent heavy metal contaminant poses serious risks to plants, humans, and the environment. The ubiquity of this toxic metal is continuously increasing due to the rapid discharge of industrial and mining effluents and the excessive use of chemical fertilizers. Nanoparticles (NPs) have emerged as a novel strategy to alleviate Cd toxicity. Zinc oxide nanoparticles (ZnO-NPs) have become the most important NPs used to mitigate the toxicity of abiotic stresses and improve crop productivity. The plants quickly absorb Cd, which subsequently disrupts plant physiological and biochemical processes and increases the production of reactive oxygen species (ROS), which causes the oxidation of cellular structures and significant growth losses. Besides this, Cd toxicity also disrupts leaf osmotic pressure, nutrient uptake, membrane stability, chlorophyll synthesis, and enzyme activities, leading to a serious reduction in growth and biomass productivity. Though plants possess an excellent defense mechanism to counteract Cd toxicity, this is not enough to counter higher concentrations of Cd toxicity. Applying Zn-NPs has proven to have significant potential in mitigating the toxic effects of Cd. ZnO-NPs improve chlorophyll synthesis, photosynthetic efficiency, membrane stability, nutrient uptake, and gene expression, which can help to counter toxic effects of Cd stress. Additionally, ZnO-NPs also help to reduce Cd absorption and accumulation in plants, and the complex relationship between ZnO-NPs, osmolytes, hormones, and secondary metabolites plays an important role in Cd tolerance. Thus, this review concentrates on exploring the diverse mechanisms by which ZnO nanoparticles can alleviate Cd toxicity in plants. In the end, this review has identified various research gaps that need addressing to ensure the promising future of ZnO-NPs in mitigating Cd toxicity. The findings of this review contribute to gaining a deeper understanding of the role of ZnO-NPs in combating Cd toxicity to promote safer and sustainable crop production by remediating Cd-polluted soils. This also allows for the development of eco-friendly approaches to remediate Cd-polluted soils to improve soil fertility and environmental quality.

## 1. Introduction

**Figure 1:**
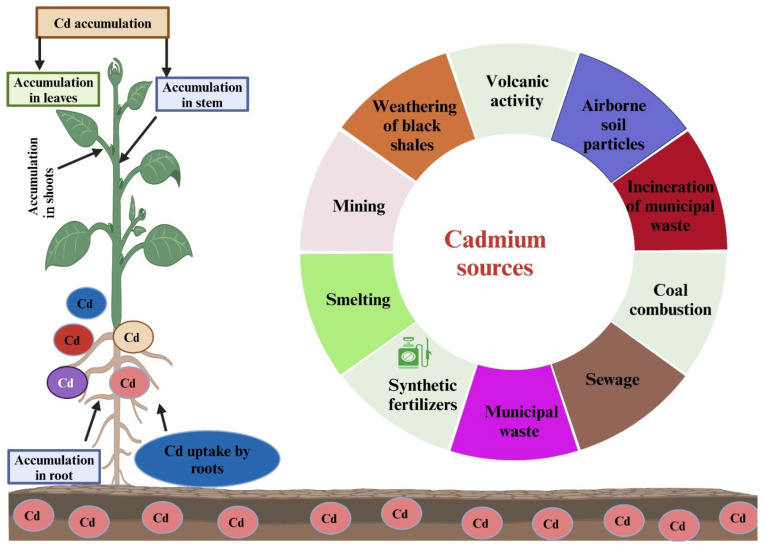
Cadmium enters into soils through different anthropogenic and geogenic sources, which causes toxicity in plants and humans.

Heavy metals (HMs) pollution is a serious challenge across the globe due to their toxic impacts on plants, humans, animals, and environmental quality [1]. The concentration of HMs is continuously increasing in soil and water, which needs to be addressed urgently for safer food production and global food security [2]. Different HMs accumulate in soil due to industrial waste and sewage disposal. An excessive concentration of Cd negatively impacts crop growth and development, leading to reduced crop productivity. Moreover, cadmium can enter the human food chain by using Cd-contaminated food, which poses a significant health hazard to individuals [3]. Cadmium (Cd) is a toxic and non-essential metal for plants and humans, and its concentration is significantly increasing in environment due to its wide use in different industries. It is considered among the three most dangerous pollutants, along with lead (Pb) and mercury (Hg) [4]. The concentration of Cd in non-contaminated soils can vary from 0.1–0.5 mg kg^−1^; nevertheless, human activities (Figure 1) have increased Cd concentration to more than tolerable limits [5]. Cadmium can cause kidney stones, renal tube damage, emphysema, liver damage, and reduced calcium (Ca) supply to the human body [6,7].

The transfer of Cd into the soil and the environment results from both natural processes and human activities. Amid natural sources, rock weathering, sea spray, forest fires, wind dust (Figure 1), and volcanic eruptions are major sources of Cd entry into the environment [8]. On the other hand, human activities like cement industries, sewage sludge, traffic, power stations (Figure 1), waste water, waste incinerators, and battery industries are major sources of Cd entry into the environment [9]. Over the past decade, there has been a notable increase in the release of Cd into the environment, leading to significant global concerns. The research conducted by Yuan et al. [10] revealed that 90.4% of Cd emissions from human activities are released into water bodies.

Cadmium is highly toxic to plants, and despite this toxicity, plants exhibit a rapid rate of absorption of Cd due to its availability in the environment [7]. Roots are the primary way of entry for Cd in plants, and different metal channels and transporters play a critical role in Cd uptake from roots to their transportation in aerial plant parts [11]. Cadmium toxicity in plants causes diverse changes ranging from disruption in cell division, plant growth, photosynthetic efficiency, chlorophyll synthesis, enzyme activity, and increase in oxidative stress [12,13,14]. Cd-induced oxidative stress also causes membrane damage, disturbs electron transport, and interferes with nucleic acids (DNA) and photosynthesis [15,16]. It also inhibits carbon fixation, lateral root growth, and stomata density and causes substantial growth losses [17,18]. Additionally, it triggers osmotic stress by decreasing leaf relative water content (RWC), stomatal conductance, and transpiration rate [19], and diminishes nutrient uptake, consequently leading to substantial yield losses [20].

Cadmium toxicity detrimentally impacts plant processes, producing increased reactive oxygen species (ROS). Nonetheless, plants have evolved an impressive antioxidant defense system to mitigate the effects of Cd toxicity [21]. Plants also synthesize various osmolytes to counteract the toxic impacts of Cd [22], and they also chelate and sequester Cd and enhanced gene expression to counter Cd toxicity [22]. The conventional approaches like lime stabilization/solidification, hydroxide compound application, solvent extraction, and in situ remediation strategies are insufficient in mitigating Cd toxicity. However, emerging fields such as nanotechnology shown promising outcomes in addressing Cd toxicity. Nanotechnology involves using nanomaterials ranging from 1–100 nm, and these materials improve crop productivity and stress tolerance, owing to their excellent surface area and reactivity [23,24]. The application of NPs is a multi-faceted approach to support plant growth, nutrient uptake, and photosynthesis and protect plants from oxidative stress [23]. Zinc oxide nano particles (ZnO-NPs) are commonly used NPs with a large surface area, catalytic activity, and chemical and physical behaviors [25]. Applying ZnO-NPs has shown promising results against drought, salinity, and heavy metals toxicity. ZnO-NPs enhance plant growth, biomass production, chlorophyll synthesis, nutrient uptake, and water absorption while mitigating Cd-induced oxidative damage, promoting overall plant vigor [26,27]. Zinc oxide nanoparticles also reduce the accessibility of Cd and restrict the uptake and accumulation of Cd in plants [28]. Moreover, ZnO-NPs also increase antioxidant activities, which reduces Cd-induced oxidation and increases the synthesis of organic acids (citric acid, malic acid, and maleic acid) and amino acids (arginine, glutamate, and phenylalanine), which alleviates Cd toxicity in plants [29,30].

Despite numerous studies highlighting the efficacy of ZnO-NPs in mitigating the toxic impacts of Cd and improving crop productivity [26,31], a comprehensive review specifically elucidating the mechanisms underlying ZnO-NPs’ Cd mitigation remains lacking. Consequently, concerted efforts are imperative to investigate these mechanisms and ensure food safety amid escalating Cd pollution. This review delves into the sources of Cd contamination and its absorption and accumulation mechanisms, while simultaneously exploring the potential of ZnO-NPs in mitigating cadmium toxicity. Furthermore, it addresses existing research gaps and prospects for leveraging ZnO-NPs to mitigate Cd’s adverse effects, fostering safer and more sustainable food production. The insights provided in this review offer new avenues and potential solutions to combat Cd toxicity effectively through ZnO-NPs.

## 2. Methodology Used for Data Collection

Diverse research platforms such as Google Scholar, Scopus, and Web of Science provided the literature for this review. Different keywords like zinc, Zn-NPs, Cd toxicity, nanoparticles, green synthesis of Zn-NPs, NPs, Cd stress, and ZnO-NPs alleviate Cd toxicity were used to search the literature. We used a systematic and thorough approach to collect and identify the relevant literature. This approach involved searching different findings with special emphasis on peer-reviewed sources, global studies, and the inclusion of studies from 2000. In total, we used 171 peer-reviewed studies to write the present review article. Moreover, we only considered studies published in English, and studies in other languages were excluded from the analysis.

## 3. Cadmium Toxicity, Uptake, and Translocation in Plants

Cadmium pollution is a serious concern globally owing to its persistence in soil for a long period [4]. Globally, around 41,000 tons of Cd is emitted into the soil every year [32], and it has a half-life of 10–33 years, leading to longer soil persistence [4]. The concentration of Cd is increasing in soil and environment due to anthropogenic activities. Therefore, Cd toxicity is becoming a huge challenge for crop productivity and food security.

Plant roots are primary pathway for Cd uptake; however, leaves can absorb Cd through foliar spray. In plants, the mechanism of Cd uptake can be differentiated into four sub-stages: uptake by roots, xylem loading, Cd transport to shoots, and the phloem translocation of Cd into seeds [33]. However, different factors like soil pH, soil texture, clay concentration, soil organic matter (SOM), cation exchange capacity (CEC), and hydrous oxidase affect the Cd bioavailability [34]. In soils, Cd can also become mobile due to its weak binding with exchange sites, SO, carbonate, and hydrous oxides [35]. This characteristic plays a critical role in increasing the bioavailability of Cd for plants. The elevated soil salt concentrations also enhance Cd mobility, resulting in increased Cd uptake and toxicity [36]. Further, moist soil conditions also increase soluble Cd concentration in soil and facilitate its absorption and subsequent translocation in plants [37].

Due to their physio-chemical characteristics, Cd and plant nutrients vie for similar transporters [38]. Thus, Cd uptake, transport, and distribution in plants involve plasma membrane transporters that are involved in the uptake of calcium (Ca), Cu, iron (Fe), Mn, and zinc (Zn) [39]. Generally, roots absorb Cd as divalent Cd (Cd^2+^), which enters the roots using different transporters [40]. Phytosiderophores also chelate Cd, resulting in the formation of Cd–phytosiderophore complexes. It is documented that different transporters like YSL2 and YSL3 play an important role in the uptake of these Cd–phytosiderophore complexes across the root plasma membrane [41]. A significant proportion of Cd is transported into root cells after absorption, while only a minimal amount of Cd is transferred into shoots and grains [42].

Plant root cells absorb Cd and are transported into xylem vessels for long-distance transport. Generally, plants use symplastic and apoplastic passages to transport Cd to xylem vessels [43]. Subsequently, Cd traverses Casparian strip barriers, which impede solute movement through cell walls, necessitating the active transport of Cd into the xylem. After loading in the xylem, Cd is moved with a transpiration stream to reach the upper plant parts [44]. Cd is absorbed into leaf tissues by cuticle and epidermal cells [45], and then it is transported into plant parts and accumulates in different tissues. Indeed, the mechanism of Cd uptake is not fully understood, and it can vary depending on factors such as Cd speciation, leaf characteristics, and environmental conditions. It is known that foliar Cd uptake contributes less to the overall Cd absorption than root uptake. However, foliar uptake becomes more significant in conditions of higher Cd deposition and limited root uptake. Cd toxicity negatively impacts plants, highlighting the importance of preventing its entry into plants to protect both plant and human health.

Cadmium induces seriously toxic impacts on plants and causes leaf necrosis and chlorosis and disrupts the photosynthetic activity and enzyme activity involved in the Calvin cycle. It also damages the photosynthetic system, thereby causing a reduction in plant growth and development [46]. It also decreases the uptake of essential nutrients (Fe and Mg) that play a significant role in chlorophyll synthesis [47]. Thus, Cd-induced nutrient deficiency negatively affects plant physiological functioning, resulting in significant growth reduction [48]. In addition, Cd-induced oxidative damage causes the oxidation of cellular structure, DNA, and protein and negatively affects the plant’s physiological and biochemical functioning [49].

## 4. Synthesis of Zinc Oxide Nanoparticles

**Figure 2:**
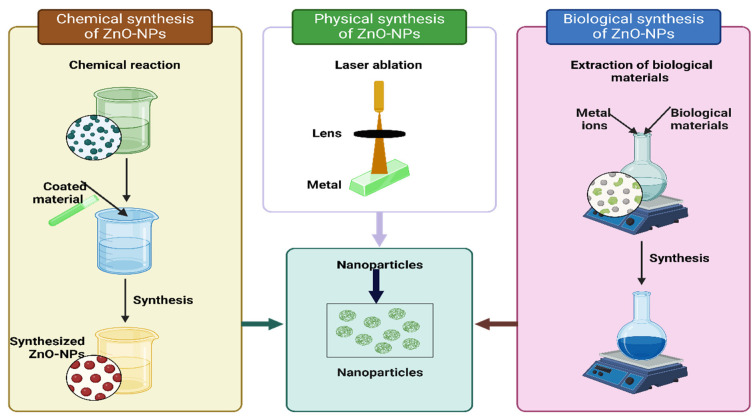
Different methods of used for the synthesis of ZnO-NPs.

Different biological, chemical, and physical methods are used around the globe to synthesize Zn-NPs (Figure 2). The chemical method is an important method used globally to synthesize ZnO-NPs, and it can be used in diverse temperature conditions and reactant concentrations. The vapor transport method is a commonly used chemical method; in this method, nanostructures of ZnO are produced in the reaction of oxygen and zinc. The vapors of Zn can be produced through different methods; for example, zinc powder can be heated in the presence of oxygen at a moderate temperature to produce zinc vapor. However, oxygen pressure to vapor pressures must be carefully handled for the appropriate production of ZnO-NPs [50]. This is an important and effective method to control the size of NPs, owing to the low temperature used in synthesis processes. Moreover, this technique uses a simple apparatus and catalyst-free growth, and it is also less toxic, economical, and eco-friendly [51].

Different green methods are also used globally to synthesize ZnO-NPs. These methods involve the use of algae, bacteria, fungi, and plants to synthesize ZnO-NPs [51,52]. Green synthesis is a rapid, eco-friendly, and expensive method to produce ZnO-NPs [53]. Green-synthesized ZnO-NPs have shown promising results in mitigating the toxic effects of plant stress conditions [54]. Various biological systems are utilized for NP production; however, employing microbes for NP synthesis poses challenges due to the intricate requirements for intracellular production and cell culture maintenance. Therefore, in this context, using plant materials has emerged as a less expensive and eco-friendly way to produce more NPs commercially [55]. This technique includes thoroughly washing plant parts, sanitizing them, rinsing them under running water, and then drying and grinding the plant parts. The resulting powder is mixed with Milli-Q water and cooked by stirring to prepare NPs. The solution is filtered afterward, and the obtained extract can be heated with zinc at the proper temperature to produce ZnO-NPs [56]. Besides this, ZnO-NPs can also be produced by laser vaporization, co-precipitation, ball milling, and the sol-gel process [57]. However, these methods are costly and need equipment, stabilizers, and toxic chemicals [54].

## 5. Mechanisms of Zinc Nanoparticles’ Uptake and Transport in Plants

Foliar spray and root application are widely used methods for applying NPs to plants [58]. After entering the plant tissues, nanoparticles enter the cell wall and cell membranes of the cortex and epidermis and undergo different events to enter the plant xylem [59,60]. Root hair cells absorb the nanoparticles, which are then transported through the epidermis and xylem vessels and then reach the underground parts [61]. In foliar spray, ZnO-NPs are applied to the leaf surface and then they are absorbed by leaf cuticle and stomata. As a result, nanoparticles are transported within the plants through phloem sieve tubes [62]. For instance, Zhu et al. [63] reported that ZnO-NPs penetrate the leaf epidermis through stomata, accumulate in the plastid, and translocate into chloroplasts. However, it is important to consider the size of ZnO-NPs to prevent stomata blocking [64] because thick leaf cuticles can also serve as a barrier to the entry of NPs [65].

Different soil characteristics like pH, SOM, humic acid, salinity, and soil clay play a critical role in the transportation and retention of metals. ZnO nanoparticles have a tendency to aggregate in soil, forming interactions with soil particles, which can decrease their diffusivity and reactivity [66,67]. The dissolution of ZnO-NPs is an important factor in evaluating their migration, toxicity, and availability to plants and microbes. However, dissolution depends on the properties of NPs, planting species, and soil characteristics [68]. For instance, SOM can increase the mobility of ZnO-NPs by decreasing the retention time [69]. Soil pH and humic acid also affects the dissolution and aggregation of ZnO-NPs. Further, ZnO-NPs with positive charge can absorb negatively charged humic acid under lower soil pH, leading to their aggregation [70]. The size of NPs also affects their dissolution; for instance, small-sized NPs have quick dissolution compared to large-sized NPs [71]. In addition, soil acidity and alkalinity also affect the dissolution of ZnO-NPs; for example, ZnO-NPs are more quickly absorbed in acidic soils than in alkaline ones [72].

The size of NPs also plays an important role in the uptake of NPs; for instance, large-sized NPs can face a problem when entering cell walls and cell membranes [73], while small-sized ZnO-NPs can easily pass the cell wall and cell membranes [73].

## 6. Mechanisms of Zinc Oxide Nanoparticles to Mitigate Cd Toxicity

**Figure 3:**
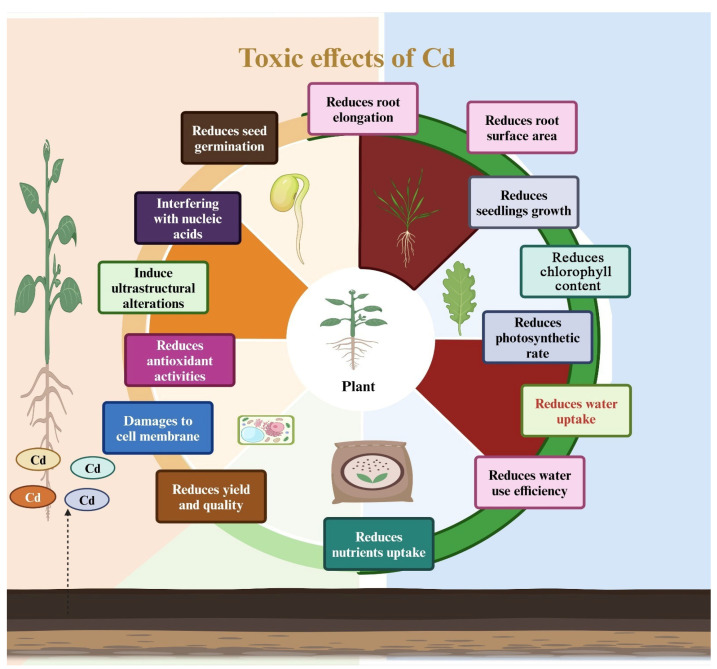
The mechanisms of cadmium-induced toxic impacts on plants.

Cadmium stress produces toxic plant effects, including growth inhibition, reduced biomass production, and negative impacts on photosynthesis, water, and nutrient uptake (Figure 3). It also increases oxidative stress and causes ultrastructural damage, resulting in substantial growth losses. While applying ZnO-NPs shows promise in alleviating Cd’s adverse effects, the exact mechanisms by which ZnO-NPs mitigate Cd toxicity remain unclear. Therefore, we have outlined the most recent understanding of the mechanisms by which ZnO-NPs alleviate Cd toxicity in plants.

### 6.1. Zinc Oxide Nanoparticles Improve Photosynthetic Efficiency under Cd Toxicity

Photosynthesis stands as one of the most vital processes in plants; however, Cd toxicity detrimentally impacts this process, leading to a significant reduction in plant growth (Table 1). Faizan et al. [74] reported that Cd toxicity reduced the photosynthetic rate (Pn), stomata conductance (gs), CO_2_ concentration (Ci), and transpiration rate (Tr) of rice (*Oryza sativa* L.) plants by 40%, 31.67%, 38.46%, and 29.09%. However, ZnO-NPs (50 mg/L) reduced the toxic impacts of Cd and improved the photosynthetic attributes [74]. Various authors have observed an increase in chlorophyll synthesis after applying ZnO-NPs. This increase has been associated with the stabilization of the photosynthetic apparatus, along with a reduction in oxidative damage and Cd absorption with ZnO-NPs [75]. Recently, Hussain et al. [76] also found that ZnO-NPs (50 mg L^−1^) significantly enhanced chlorophyll and carotenoid synthesis under Cd stress, which ensured better synthetic efficiency, subsequently accelerating production in plant growth [14]. Heavy metals like Cd produce excessive ROS that damage the photosynthetic apparatus and reduce chlorophyll synthesis by increasing the activity of chlorophyll-degrading enzymes [74,77]. Zinc nanoparticles play a protective role in safeguarding the photosynthetic apparatus, as they reduce the activity of chlorophyll-degrading enzymes, leading to enhanced photosynthetic efficiency [74]. Gao et al. [78] recently reported that ZnO-NPs at a concentration of 2.5 mg L−^1^ showed a significant increase in chlorophyll a (26%) and chlorophyll b (30%) contents in lettuce (*Lactuca sativa* L.) seedlings. The observed increase in photosynthetic efficiency and chlorophyll synthesis was attributed to maintaining membrane stability, cell viability, photosynthetic apparatus, nutrient uptake, gene expression, and a reduction in oxidative damage.

Zinc nanoparticles are combined with other amendments to improve their efficiency against Cd stress. For instance, the application of ZnO-NPs (50 and 100 mg/kg) increased chlorophyll synthesis (18% and 34%), chlorophyll b contents (26% and 55%), and carotenoid contents (16.4% and 47.5%). However, the combined use of ZnO-NPs and melatonin (MT) showed more promising results, and the application of 100 mg/kg ZnO-NPs and 100 μM MT increased chlorophyll-a, chlorophyll-b, and carotenoid concentrations by 63.5%, 103.8%, and 153.3%, respectively [99]. The increase in photosynthetic efficiency following ZnO-NPs is linked with an adequate supply of Zn, which stimulates chloroplast density and bioactivity and increases chlorophyll synthesis [100].

Chen et al. [101] recently discovered genes linked with porphyrin and chlorophyll metabolism pathways by applying ZnO-NPs. This indicates that ZnO-NPs, Cd, and their combined use can affect photosynthesis. These expression profiles of down-regulated chloroplast genes showed a higher expression in the presence of Cd stress with the application of ZnO-NPs. This indicates that ZnO-NPs activate rescue chloroplast genes, thereby producing better chlorophyll synthesis under Cd stress [101]. These studies suggest that the enhanced photosynthetic efficiency associated with ZnO-NPs was correlated with improved antioxidant activity and the reduced availability and toxicity of Cd. Cd toxicity hampers chlorophyll synthesis by impeding the absorption of macro- and micronutrients essential for the photosynthetic machinery. However, applying ZnO-NPs leads to increased nutrient uptake and reduces damage caused by ROS to the photosynthetic apparatus, significantly enhancing photosynthetic efficiency.

### 6.2. Zinc Oxide Nanoparticles Improve Membrane Stability, Plant Water Relation, and Nutrient Uptake under Cd Toxicity

Cadmium toxicity damages the membranes by increasing ROS and MDA production. ZnO-NPs showed promising results in mitigating Cd toxicity and ensuring better membrane stability under Cd stress. For example, Shafiq et al. [102] stated that ZnO-NPs, in combination with plant growth promoting rhizobia (PGPR), improved the RWC by 248% and membrane stability in Cd-treated maize (*Zea mays* L.) plants [102]. Cd stress is known to cause membrane damage, resulting in reduced relative water content (RWC) and increased levels of MDA and electrolyte leakage (EL). A recent study highlighted that applying ZnO-NPs reduced MDA and EL levels by increasing antioxidant activities and the synthesis of osmolytes [101].

Cadmium toxicity disrupts nutrient uptake [103] by interfering with the absorption and transport of minerals, resulting in reduced nutrient accumulation and inhibiting plant growth [104]. Applying ZnO-NPs (50 and 100 mg/kg) under Cd stress increased the uptake and accumulation of Zn in roots, shoots, and husks as compared to their lone application. The exogenic ZnO-NPs particularly (100 mg/kg) increased root, shoot, and husk Zn concentration by 28.2%, 41.4%, 51.1%, and 35.2% [99]. This indicates that the ZnO-NP application rate is crucial in improving Zn concentration in plant parts. In another study, Cd toxicity induced a significant decrease in Cu, Ca, Zn, K, Mg, Cd, Fe, and sodium (Na) concentration; however, ZnO-NPs increased the concentration of these nutrients from 100–458% under Cd stress [102].

Zinc nanoparticles applied to tomato (*Solanum lycopersicum* L.) plants increased the leaf Cu, Mn, and Zn concentrations by 13%, 60.8%, and 78.6%, while ZnO-NPs increased root Cu, Mn, and Zn concentrations by 267.8% and 33.9%. This suggests that ZnO-NPs enhance nutrient uptake, promoting better plant growth [72]. Perilla (*Perilla frutescens* L.) seedlings that received ZnO-NPs under Cd stress showed an increase in Fe and Zn in leaves along with Cu and Mn in roots [105]. Similarly, applying ZnO-NPs to tobacco (*Nicotiana tabacum* L.) plants increased Cu, Mg, and K concentrations [106]. This suggests that ZnO-NPs enhance nutrient uptake, although the precise absorption mechanism remains unclear. Therefore, there is an urgent need for further studies to investigate the mechanisms underlying nutrient uptake and accumulation following ZnO-NP application. ZnO-NPs also increased the Zn concentration in root, shoot, and grain, decreasing the Cd concentration in green peas (*Pisum sativum* L.) [107]. The higher concentration of Zn in plants can decrease the concentration of Cd [108]. Wheat (*Triticum aestivum* L.) plants supplemented with the soil application of ZnO-NPs (25 mg kg^−1^) showed a marked increase in Zn concentration and reduced Cd concentration [23]. This suggests that an increase in Zn uptake leads to a decrease in Cd concentration. Nevertheless, additional studies are needed to clarify the mechanisms by which ZnO nanoparticles reduce Cd uptake in wheat. These findings indicate the potential of ZnO-NPs to maintain membrane stability and plant water balance under Cd stress. Additionally, ZnO-NPs improve nutrient uptake, possibly linked to the enhanced gene expression of metal transporters. These discoveries also offer significant potential in understanding the effects of ZnO-NPs on nutrient signaling, channels, and gene expression associated with metal transporters. Moreover, it is crucial to assess the impact of different concentrations of ZnO-NPs on nutrient uptake and accumulation in various plant species. Understanding the molecular mechanisms of ZnO-NP-mediated increase in nutrient uptake could pave the way for novel strategies to enhance crop production while mitigating Cd toxicity.

### 6.3. Zinc Oxide Nanoparticles Strengthen Antioxidant Defense System to Counteract Cd Toxicity

The excessive accumulation of Cd in plants results in the overproduction of ROS, which can cause membrane damage and increased MDA production [109]. Numerous studies have shown that ZnO-NPs improve antioxidant activities to counter Cd toxicity (Table 2). For instance, in melon (*Cucumis melo* L.) plants, Cd toxicity increased superoxide dismutase (SOD: 46.42%), catalase (CAT: 41.19%), and peroxidase (POD: 46.51%) activities as compared to control. This indicates that melon activated its antioxidant defense system to counter Cd toxicity. Further, the application of ZnO-NPs in combination with *B. fortis* IAGS enhanced SOD, CAT, and POD activities by 86.84%, 44.47%, and 6.86% as compared to the sole application of ZnO-NPs. The synergistic use of ZnO-NPs and *B. fortis* IAGS showed more promising results in improving the antioxidant activities, which helped to counter the Cd-induced toxicity [110]. Likewise, another study conducted on rice showed that Cd toxicity increased H_2_O_2_ and MDA production by 34% and 29%. The supplementation of ZnO-NPs under Cd toxicity reduced MDA and H_2_O_2_ production by 73% and 57%, respectively [74]. The application of ZnO-NPs also increased antioxidants under both normal and Cd stress conditions, and maximum CAT (52%) and SOD (59%) activity was observed in plants treated with ZnO-NPs + Cd [74].

**Figure 4:**
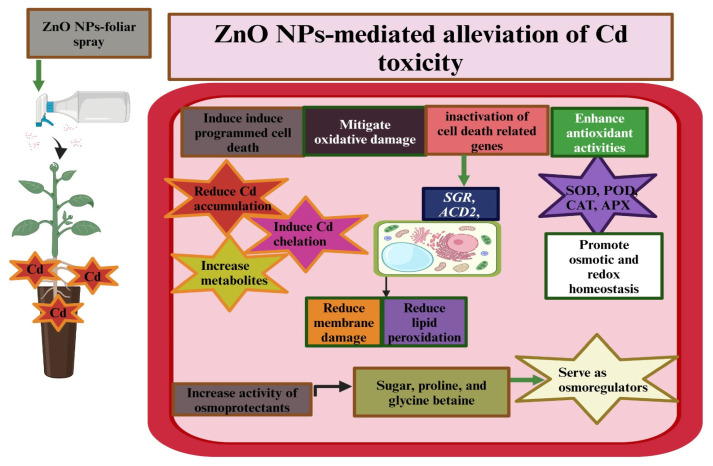
A model showing the mechanisms of ZnO-NPs in alleviating Cd accumulation and its toxic impacts on plants.

ZnO-NPs mediated the increase in antioxidant activities (APX, CAT, POD, and SOD: Figure 4), which substantially improved wheat and maize growth under Cd toxicity [23,111]. This indicates that applying ZnO-NPs to Cd-stressed plants improved antioxidant activities, which reduced ROS production. Applying ZnO-NPs to wheat plants under Cd stress enhanced the APX, CAT, AsA, GSH, POD, and SOD activities, which reduced Cd-induced oxidative burst [112]. Using ZnO-NPs in alfalfa (*Medicago sativa* L.) plants boosted antioxidant activity and decreased oxidative damage [101]. This implies that ZnO-NP application strengthens the antioxidant defense system, which helps counter the toxic effects of Cd (Figure 4). Further, ZnO-NPs also increase the expression of peroxidase genes under Cd, increasing POD activity and ensuring the plants tolerate Cd-induced oxidative bursts [101]. GSH is a non-protein thiol that plays a pivotal role in mitigating Cd toxicity due to the strong affinity of its thiol group [113]. The exogenous supply of Zn-NPs has been reported to increase GSH activity, which has helped maintain redox balance in Cd-polluted conditions [101]. Lopez-Moreno et al. [114] found an increase in antioxidant activities under ZnO-NP application, which resulted from gene expression and its involvement in diverse oxidative processes [115,116]. Another group of authors also reported that ZnO-NPs reduced MDA, H_2_O_2_, and O_2_ concentrations under Cd toxicity in wheat, rice, maize, and tomato, which was a reflection of the enhanced activities of APX, CAT, POD, and SOD [74,111,117].

**Table 2:** Effect of ZnO-NPs on growth, physiological functioning, nutrient homeostasis, antioxidant activities, and Cd uptake in different plant species.

**Plant Species**	**Mode of Application**	**Growth Conditions**	**Cd Concentration**	**Major Effects**	**References**
*Oryza sativa*	Foliar spray (0.05%)	Field	0.85 mg kg^−1^	Zn-NPs increased rice yield and grain zinc concentration and reduced Cd concentration in plant parts and its bio-accessibility.	[100]
*Pennisetum giganteum*	Soil application (50 and 100 mg kg^−1^)	Pot	40 mg kg^−1^	ZnO-NPs improved the antioxidant response, reduced oxidative damage, increased soil enzyme activities (catalase, saccharase, and urease), and enriched microbial communities at low concentration.	[118]
*Brassica oleracea*	Seed priming (50 and 100 mg kg^−1^)	Pot	25 and 50 mg L^−1^	The application of 50 and 100 mg L^−1^ ZnO-NPs increased the growth by 38% and 40% with substantial increases in Zn concentration by 20.1% and 24%. Further, ZnO-NPs also increased chlorophyll synthesis, anthocyanin, flavonoid, soluble sugars, protein, free amino acids, APX, and CAT activity and reduced EL.	[119]
*Lactuca sativa*	Foliar spray (10 and 100 mg L^−1^)	Pot	0, 2, and 10 mg kg^−1^	Foliar spray of NPs reduced the Cd uptake and maximum reduction (43%) was seen with application of 100 mg L^−1^ NPs. Further, the same treatment also increased chlorophyll synthesis, CAT, and SOD activity and Zn concentration.	[120]
*Brassica parachinensis*	Foliar spray (50 and 100 mg L^−1^)	Pot	10 μM	Foliar feeding of NPs increased plant height, root growth, biomass production, nutrient uptake (Cu, Fe, Zn and Mg), photosynthetic pigments, POD, CAT, and SOD activities and reduced MDA production. Further, ZnO-NPs also restored 60% of Cd stress metabolites into normal leaves.	[121]
*Triticum aestivum*	Foliar spray (75 and 100 mg L^−1^)	Field	0.5 mg kg^−1^	ZnO-NPs increased the grain yield (14% and 15%) and reduced grain Cd (23.8% and 33.6%), bioaccessibility and daily intake of Cd, and uptake of Ca, S, Fe, B, and zinc.	[122]
*Capsicum annuum*	Foliar spray (100 mg L^−1^)	Pot	100 mg kg^−1^	ZnO-NPs mitigated the adverse impacts of Cd and improved root and shoot growth, soluble carbohydrates, proteins, amino acids, and photosynthetic pigments.	[123]
*Zea mays*	Foliar spray (0, 50, 75, and 100 mg L^−1^)	Pot	4.15 mg kg^−1^	The exogenic spray of NPs enhanced maize growth, leaf gas exchange properties, photosynthesis, and antioxidant activities (APX, CAT, POD, and SOD) and decreased MDA and H_2_O_2_ production. Further NPs (100 mg L^−1^) also decreased the shoot (74.55), root (66.38%), and grain Cd (76.19%).	[124]
*Vigna radiata*	Nutrient solution (0, 1, 2, 4, 8, 16, and 32 μM)	Hydroponic	0, 0.5, and 1 μM	The exogenous supply of ZnO-NPs improved photosynthetic pigments, biomass production, and APX, CAT, POD, and SOD activities. Further NP application also enhanced root, stem, and grain Zn concentration by 33-, 10-, and 17-fold, respectively, while NPs also reduced Cd concentration in plant parts.	[125]
*Oryza sativa*	Nutrient solution (5 and 10 mg L^−1^)	Hydroponic	2.26 mg L^−1^	ZnO-NPs improved nitrogen and protein in root and aerial plant parts and increased the expression of myeloblastosis, zinc-finger protein, and ascorbate peroxidase genes.	[126]
*Phytolacca americana*	Soil application (0 and 500 mg kg^−1^)	Pot	10 and 100 mg kg^−1^	The application of ZnO-NPs improved growth, development, and Cd translocation to plant plants and improved antioxidant activities accompanied by a two-fold increase in thiobarbituric acid reactive substances.	[127]
*Oryza sativa*	Seed priming (0, 25, 50, and 100 mg L^−1^)	Pot	0 and 100 mg L^−1^	ZnO-NPs increased the shoot fresh weight by 16.92–27.88%, POD, SOD, and metallothionein. Further, NPs also increased α-amylase and total amylase activity, Zn concentration, synthesis of alanine, aspartate and glutamate metabolism, and phenylpropanoid.	[128]
*Triticum aestivum*	Foliar spray (25 mg L^−1^)	Pot	4.23 mg kg^−1^	ZnO-NPs increased straw and grain yields, chlorophyll and carotenoid synthesis, and antioxidant activities and reduced the EL and reduced the Cd uptake in straw and grain by 84% and 99%, respectively.	[129]
*Triticum aestivum*	Foliar spray (0, 100, and 200 mg L^−1^)	Pot	7.65 mg kg^−1^	The application of ZnO-NPs (200 mg L^−1^) increased biomass production, yield, chlorophyll synthesis, and POD and SOD activities and reduced the concentration of Cd in roots, shoots, and husks.	[7]
*Triticum aestivum*	Soil application (25, 50, and 100 mg kg^−1^)	Pot	7.67 mg kg^−1^	Soil-applied NPs increased yield, chlorophyll synthesis, and antioxidant activities and reduced MDA, H_2_O_2_, and EL production.	[130]
*Oryza sativa*	Soil application (50, 100, and 500 mg kg^−1^)	Pot	1, 2.5 and 5 mg kg^−1^	The addition of ZnO-NPs increased soil pH and increased the plant height and biomass by 13–22% and 25–43% under medium and higher Cd stress. The higher concentration of NPs (500 mg kg^−1^) increased Cd bioavailability (0.51 mg kg^−1^) exceeding the acceptable limit (0.2 mg kg^−1^).	[131]
*Oryza sativa*	Foliar spray (0, 50, 75, and 100 mg L^−1^)	Pot	7.86 mg kg^−1^	ZnO-NPs (100 mg L^−1^) improved biomass production and photosynthesis and diminished the Cd root and shoot levels by 31% and 30%, respectively and reduced soil AB-DTPA extractable Cd concentration.	[132],
*Portulaca oleracea*	Foliar spray (50 and 100 mg L^−1^)	Pot	50 and 100 μM	Foliar spray of ZnO-NPs improved antioxidant activities in the glyoxalase system and reduced MDA, H_2_O_2_, and EL production. Further, ZnO-NPs improved photosynthetic pigments, decreased Cd uptake, and increased leaf micro- and macro-nutrients’ concentration.	[133]
*Sorghum bicolor*	Soil application (50, 250, and 500 mg kg^−1^)	Pot	5 mg kg^−1^	The application of lower doses of NPs (50 mg kg^−1^) increased plant growth, soil enzymes activities, nutrient uptake, and antioxidant activities and reduced Cd uptake. However, a higher dose of NPs inhibited plant growth and led to synergistic toxicity along with Cd.	[134]
*Oryza sativa*	Foliar spray (100 mg L^−1^)	Pot	5 mg kg^−1^	Foliar spray of NPs increases the plant growth, biomass production, and Fe and Cu uptake and reduced the Cd accumulation in plant parts.	[135]
*Capisum annum*	Foliar spray (2.5, 5, and 10 mg L^−1^)	Pot	200 μM	Exogenous supplementation of ZnO-NPs improved shoot length (70–85%), shoot weight (40–50%), length (60–80%), CAT (30–75%), and glutathione peroxidase (13–63%) and reduced the MDA, H_2_O_2_ production, and Cd uptake.	[136]
*Spinacia oleracea* and *Portulaca oleracea*	Nutrient solution (100 mg L^−1^)	Hydroponic	100 mg L^−1^	The use of NPs enhanced growth, biomass production, and Cu and Fe accumulation in plant parts and reduced the uptake, accumulation, and bioavailability of Cd.	[137]
*Vicia faba*	Foliar spray (250, 500, and 1000 mg L^−1^)	Pot	25 and 50 mg L^−1^	The application of ZnO-NPs increases SOD, glutathione reductase (GR), glutathione peroxidase (GPX), CAT, and glutathione (GSH) activity and reduced MDA and H_2_O_2_ production and led to a significant reduction in Cd toxicity.	[138]
*Nicotiana tabacum*	Foliar spray (50 mg L^−1^)	Pot	50 mg L^−1^	Foliar spray of NPs clearly increased plant height, root and shoot growth, zinc, K, and Mn uptake, and synthesis of amino acids, arginine, proline, flavone, flavonol, nicotinate, and nicotinamide.	[106]
*Lactuca sativa*	Seed priming (100 mg L^−1^)	Pot	5 μM	NPs’ seed priming effectively reduced MDA and H_2_O_2_ production, improved root morphology and nutrient uptake (Ca, Fe, K, Mn and Zn), and markedly reduced the uptake and accumulation of Cd.	[139]
*Triticum aestivum*	Soil application (0, 150, and 300 mg kg^−1^)	Pot	25 mg kg^−1^	The application of NPs (300 mg kg^−1^) increased shoot weight (66%), root weight (58%), grain yield (137%), and Zn concentration (58%) and decreased Cd (33%) concentration. Further, NPs also reduced Cd accumulation in root, shoot, and husks and resulted in minimum bioaccumulation (0.14), health risk (0.05), and translocation index (2.46).	[140]

The effects of ZnO-NPs and CuO-NPs were investigated to alleviate Cd toxicity in common beans (*Phaseolus vulgaris* L.). The study observed a dose-dependent response of P. vulgaris plants against NPs at concentrations of 10, 50, 100, and 200 mg/L. The application of both NPs resulted in reduced generation of H_2_O_2_ by boosting the activity of antioxidant enzymes peroxidase (APX), CAT, glutathione peroxidase (GPOX), glutathione peroxidase (GPX), and glutathione reductase (GR) while inhibiting ROS-producing enzymes like glucose oxidase (GOX) and NADPH oxidase (NOX). Furthermore, the study revealed that another mechanistic impact of NPs involved the modulation of different enzymes (Trx, NTR, Fd, and FNR) responsible for maintaining cellular homeostasis [141]. The effect of ZnO-NPs was tested against combined Cd and Pb toxicity. The exogenic application of ZnO-NPs increased the root (182.0 and 121.1%) and shoot CAT activity by (195.2 and 180.1%) under Cd and Pb toxicity as compared to control [31]. Similarly, another study observed the impact of different concentrations of ZnO-NPs (25, 50, and 100 mg L^−1^) on rice plants. They reported an increase of 36.99, 51.37, and 32.79% in shoot SOD activity following the addition of different concentrations of ZnO-NPs. However, no significant change in shoot SOD activity was observed in normal conditions. On the other hand, 50 and 100 mg L^−1^ ZnO-NP applications increased POD activity by 36% and 39%, respectively [120]. The studies mentioned above provide evidence that ZnO-NPs activate the antioxidant defense system, aiding in the mitigation of ROS. However, the efficacy of ZnO-NPs may vary depending on their concentration, highlighting the need for further research to determine their optimal application under Cd stress conditions.

### 6.4. Zinc Oxide Nanoparticles Improve Hormonal Balance and Synthesis of Signaling Molecules under Cd Stress

ZnO-NPs exhibit a remarkable capacity to regulate osmolyte synthesis and maintain hormonal balance to combat Cd toxicity. Proline, a crucial osmolyte, experiences significant synthesis to counteract Cd toxicity in plants. The application of ZnO-NPs has shown promising results in enhancing proline synthesis. For instance, applying ZnO-NPs alone and in combination with *B. fortis* IAGS increased proline synthesis by 30.95% and 69.31% under Cd stress compared to the control group. The ZnO-NP-mediated increase in proline helped *the melon* seedlings to counter Cd toxicity [110]. In another study, rice plants subjected to Cd stress (0.8 mM) and ZnO-NPs showed an increase in proline synthesis. ZnO-NPs increased the concentration of proline (+17%) and soluble proteins by 38.15%, which helped the plants tolerate Cd toxicity [74]. The proline synthesis with ZnO-NPs is linked with an increase in the expression of proline biosynthesis genes [74]. Cadmium toxicity can decrease the protein concentration by increasing protease activity, which destroys the activity and structure of proteins [142]. In a very recent study, Raja et al. [143] found that the application of ZnO-NPs enhanced proline (83.94%) and glycine betaine (72.95%) synthesis, which helped the plants to maintain water balance and subsequent Cd tolerance. Likewise, Lalarukh et al. [144] found that the foliar spray of ZnO-NPs raised soluble sugars and proline, which maintained the water balance and helped wheat plants to counter Cd toxicity.

Alfalfa plants under Cd stress exhibited a significant increase in soluble sugars (SS: 92%), proline (195%), and glycine betaine (GB: 135%; [101]. Further, exogenously applied Zn also increased SS (46.77%), proline (158%), and GB (92%; [101]. These findings indicate that the application of ZnO-NPs under Cd toxicity can restore osmotic homeostasis by decreasing osmolyte accumulation and making them play a positive part [101]. In another study, it was observed that Cd and Pb stress reduced leaf protein concentration. However, applying ZnO-NPs increased total soluble proteins under Cd and Pb toxicity conditions [31]. Cadmium toxicity can increase glucose and fructose levels, and supplying ZnO-NPs (50 mg L^−1^) decreased glucose and fructose concentration but increased the sucrose concentration in both cultivars of rice [145]. Another study showed that Cd stress decreased the synthesis of flavonoids and phenolics, and the integrated use of ZnO-NPs and bacteria increased the concentration of both flavonoid and phenolics compared to their solo application [110].

The increased synthesis of phenolic compounds aids in scavenging metal ions and free radicals, thus alleviating Cd toxicity. The application of ZnO-NPs can up-regulate the synthesis of flavonoids, anthocyanins, and total phenols and up-regulate the nutrient uptake, metabolic processes, and synthesis of exudates, which ensures better plant growth under Cd stress [110]. The lettuce seedling subjected to Cd stress showed a decrease of 5% and 21% in phenylalanine (Phe) content; however, phenylalanine ammonia-lyase (PAL) activity was increased by 10% in roots and lignin concentration in roots was also increased by 18%. The exogenous application of ZnO-NPs (5 mg L^−1^) also decreased Phe concentration in leaves (5%) and roots (17%) and increased PAL activity in leaves (11%) and roots (4%) [78]. These authors also reported that ZnO-NPs (5 mg L^−1^) also increased thiol and cysteine-rich metal-binding peptides (PCs) by 9% and 38%, respectively, as compared to the control [78].

PAL plays an important role in converting phenylalanine into lignin, thereby increasing lignification. The increase in lignification increases the plastid barrier, which reduces cell wall penetration and prevents the entry of Cd [146]. Both Cd stress and ZnO-NPs can increase lignin concentration by increasing PAL and cwPOD activity, and these are key enzymes involved in the synthesis of lignin in plants [78]. Flavonoids play a critical role in plant responses to stress conditions [147]; however, Cd toxicity can decrease flavonoid synthesis [148]. Zinc oxide nanoparticles have been described as increasing the accumulation of flavonoids, and an increase in flavonoids can increase Cd tolerance in plants [149]. Cd toxicity in maize plants showed reducing effects on phenolics, flavonoids, anthocyanin, sugars, and soluble proteins. ZnO-NPs augmented the accumulation of the metabolites which helped the maize plants withstand toxic effects of Cd [76]. Applying ZnO-NPs also led to a marked increase in salicylic acid (SA) concentration in *Arabidopsis thaliana* L. leaves [150]. Additionally, Peng and Shahidi [3] discovered that tobacco plants treated with salicylic acid (SA) and ZnO-NPs exhibited enhanced photosynthetic and antioxidant activities. This suggests that ZnO-NPs may modulate endogenous SA levels, improving plant growth through strengthened antioxidant activities and regulated Cd uptake. These studies shed light on the role of ZnO-NPs in influencing signaling molecules, hormones, and secondary metabolites. However, significant gaps exist in understanding the mechanistic pathways through which ZnO-NPs enhance the synthesis of these compounds under Cd stress. Therefore, more comprehensive studies are warranted to elucidate the intricate relationships between ZnO-NPs and signaling molecules. This knowledge will facilitate the development of targeted approaches to enhance plant resilience and productivity in Cd-contaminated environments.

### 6.5. Zinc Oxide Nanoparticles Improve Genes Expression to Counter Cd Toxicity

Zn nanoparticles not only limit the uptake and accumulation of Cd but also regulate gene expression to counteract Cd toxicity. Chen et al. [101] reported that Cd and ZnO-NPs + Cd down-regulated the gene lines *Cu/Zn-SOD*, *FeSOD*, *Mn-SOD*, *CAT*, and *POD*. However, the expression of the genes above was highest in the solo application of ZnO-NPs. Gene expression plays a critical role in plant responses against Cd stress; nonetheless, the mechanisms of ZnO-NPs’ mediated increase in gene expression are still unclear. Using nanomaterials can mitigate the toxic effects of heavy metals by increasing gene expression and gene networks [151]. Zinc oxide nanoparticles can modulate the genes associated with biological and molecular processes [152]. Recently, it was documented that different heat shock protein genes (*MS.gene028507*, *MS.gene057229*, and *MS.gene26365*) were enriched in response to oxygen species and down- and up-regulated in control vs. Cd and Cd vs. Zn-NPs + Cd [101]. Another study suggests that variations in gene expression related to heat shock proteins (HSP) serve as a mechanism to defend plant against stress conditions [153]. Using ZnO-NPs can reduce Cd-induced genetic harm, chlorophyll degradation, and growth inhibition [154].

The down-regulated chloroplast-related genes showed maximum expression in control and minimum expression in Zn-NP treatment plants; however, these genes showed a relatively higher expression under ZnO-NPs + Cd [101]. These authors also found that ZnO-NPs regulate gene expression associated with antioxidant defense, resistance, and chlorophyll synthesis. Cadmium toxicity has been observed to up-regulate genes such as *SGR*, *VPE*, and *PDCD4*, which promote cell death. Conversely, ZnO-NPs down-regulate these genes, thereby reducing cell death. Furthermore, ZnO-NPs up-regulate genes like *ACD2* and *ACD11*, which inhibit cell death, ensuring plant survival under Cd toxicity conditions [101].

### 6.6. Zinc Oxide Nanoparticles Reduce Cd Uptake and Accumulation

One of the main functions of Zn-NPs is to reduce Cd toxicity by decreasing Cd uptake and transport. Studies have reported that ZnO-NPs can mitigate Cd toxicity by decreasing Cd absorption and transportation. For instance, the exogenous application of ZnO-NPs reduced the Cd concentration of various plant species [23,137]. A recent study reported that MT, in combination with ZnO-NPs, minimized the Cd concentration in plant parts, which was linked with excessive Zn that interferes with Cd’s uptake and transport in plants [99]. The decrease in Cd concentration in wheat with integrated MT and ZnO-NPs arose from the dual effects of increasing the Zn concentration and modulating antioxidant activities [99]. However, this study’s findings need thorough investigation under field conditions.

Applying Zn-NPs to wheat plants decreased the Cd concentration in roots and shoots by 15.10% and 39.77% compared to the control [74]. Roots are the first organ that come directly in contact with Cd [155], and rice plants growing under Cd showed maximum accumulation in roots. The exogenous application of ZnO-NPs reduced Cd accumulation in rice, wheat, and tomato [111,156]. These authors also claimed that the reduction in Cd concentration with ZnO-NPs was higher in areal parts than underground parts. This suggested that ZnO-NPs reduce the movement of Cd in root and shoot directions. ZnO-NPs also form the physical barriers that prevent Cd uptake, which ensures less uptake and subsequent accumulation in plant parts [74]. Recently, Lin et al. [100] also found that Zn-NPs increased straw and husk Cd concentration and decreased flag leaf and grain Cd concentration. Studies have reported that rice rachises serve as a crucial bridge for the movement of Cd and nutrients from various plant organs to grains. Following the exogenous application of ZnO-NPs, rachises transported more Cd to the husk, decreasing Cd transport from rachises to grains [100]. Hence, further studies are necessary to investigate the accumulation of Cd and Zn in various rice tissues. Additionally, transcriptomic and metabolomic studies are needed to elucidate the mechanisms underlying Cd accumulation in different plant parts following the application of ZnO-NPs.

Another study reported a similar effect of ZnO-NPs, and the authors claimed that ZnO-NPs reduced Cd absorption by rice plants and its subsequent accumulation in roots, leaves, cell walls, and cytoplasm [101]. As the concentration of Cd in the soil rises, the soil rhizosphere becomes more acidic, thereby increasing Cd availability and mobility. Nevertheless, ZnO nanoparticles decrease the absorption and accumulation of Cd in plant parts [111,154]. Plant roots allow the Cd to enter the xylem tissues, which are then transported into other tissues and cause oxidative damage. In this context, ZnO-NPs decrease Cd uptake and increase antioxidants, which help counter the toxic effects of Cd [101]. Applying Zn-NPs to autotetraploid and diploid rice plants also decreased the Cd accumulation and increased Zn accumulation [145]. This positive impact of ZnO-NPs was linked with the dissolution of NP and the release of Zn from ZnO-NPs in soil [120]. The reduction in Cd accumulation can be attributed to an increase in Zn uptake, which inhibits Cd uptake, translocation, and accumulation [157]. Besides this, ZnO-NPs also improve cell wall lignification, inhibiting Cd uptake by roots [158]. These findings imply that applying ZnO nanoparticles through different methods can reduce the uptake and accumulation of Cd in plants. Notably, foliar-applied ZnO-NPs has demonstrated greater effectiveness in reducing Cd concentrations in plant tissues. ZnO-NPs promote the immobilization of Cd and inhibit its transport through the roots, leading to a significant reduction in Cd levels within plant tissues. This highlights the potential of ZnO-NPs in mitigating Cd contamination. However, further studies are necessary to comprehensively understand the intricate interactions among ZnO nanoparticles, plants, and method of application, particularly in the context of Cd stress and different plant species. Furthermore, advanced research in this area will enhance crop production in Cd-polluted environments.

### 6.7. Zinc Oxide Nanoparticles Improve Growth under Cd Toxicity

Cadmium toxicity decreases RWC, chlorophyll synthesis, and nutrient uptake and increases ROS production, which inhibits plant growth. ZnO-NPs are an effective strategy to mitigate Cd toxicity and improve plant performance under stress conditions. For instance, foliar spray of ZnO-NPs application in rice plants mitigated the toxic effects of Cd and improved the shoot and root length by 34.0 and 25.4% and root and shoot fresh weight by 30.0% and 12.2% and their dry weights by 12.5 and 23%, respectively [74]. The mechanism behind this increase in growth was restricted Cd uptake, improved photosynthetic pigments, antioxidant activities, and nutrient uptake. The other study findings showed that ZnO-NPs applied by soil and foliar methods improved wheat growth. Though 100 mg L^−1^ ZnO-NPs application increased the dry weight of shoot and roots, number of spikes and grains by 72%, 59%, 90%, and 97%, respectively [40]. Moreover, Shafiq et al. [102] tested the impact of Zn and TiO-NPs and *Bacillus pumilus* on the growth performance of maize grown in Cd-polluted soil. Their findings revealed that nanoparticles facilitated the release of zinc and titanium to plants, thereby regulating auxin synthesis and enhancing growth by decreasing Cd uptake and Cd-induced oxidative damage.

Recently, Chen et al. [101] investigated the impact of combined application of MT and ZnO-NPs on wheat growth under Cd-polluted soil. Combining ZnO-NPs and MT enhanced root growth, nutrient uptake, photosynthesis, and antioxidant activities, ensuring better plant growth. Similarly, other authors found that soil- and foliar-applied ZnO-NPs improve wheat growth in Cd-polluted soils. This indicates that ZnO-NPs can mitigate Cd toxicity by increasing Zn uptake and reducing the Cd uptake [99]. Zinc plays an important role in the synthesis of auxin, which plays a role in cell division and cell expansion. ZnO-NPs strongly augmented the auxin synthesis, nutrient availability, and Zn uptake, ensuring better plant growth [159]. In another study, the combined application of Zn and Si-NPs stimulated the density and bioactivity of chloroplast. It increased the synthesis of chlorophyll, which in turn improved plant growth [160]. Other authors also reported a maximal increase in grain yield with co-application of Zn and Si. This indicates the synergistic impact of Zn and Si on rice growth, possibly owing to the coordinated regulation of plant biochemical and physiological function by Zn and Si [100].

The study conducted by Chen et al. [101] evaluated the response of ZnO-NPs in improving the growth of alfalfa. ZnO-NPs increased root and shoot fresh weight by 33% and 28%, linked with improved gene expression, antioxidant activities, nutrient uptake, and better photosynthetic efficiency. The study findings of Sun and his colleague showed that foliar spray of ZnO-NPs had no impact on leaf area and number of leaves; however, ZnO-NPs improved the root length and root fresh and dry biomass [148]. Similarly, Zou et al. [106] observed that ZnO-NPs mitigated Cd toxicity and enhanced plant growth and development. ZnO-NPs were found to reduce Cd concentrations in plant tissues, improve photosynthetic efficiency, and decrease ROS production, leading to improved growth and agronomic characteristics. The ability of ZnO-NPs to alleviate Cd-induced oxidative damage is attributed to their ROS scavenging properties, enhanced antioxidant activities, osmolyte accumulation, increased nutrient uptake, and reduced Cd uptake and accumulation, ultimately promoting plant growth [148]. These studies collectively underscore the potential of ZnO-NPs in enhancing crop production under Cd stress conditions.

ZnO-NPs improve crop production by enhancing photosynthetic efficiency, membrane stability, nutrient uptake, gene expression, antioxidant activities, and osmolyte synthesis and reducing Cd uptake. However, it is important to note that factors such as the concentration of ZnO-NPs and the plant species that can influence the effectiveness of ZnO-NPs to mitigate Cd toxicity.

## 7. Synergistic Role of Zinc Oxide Nanoparticles and Other Amendments to Mitigate Cd Toxicity

Globally, various amendments are applied alongside ZnO-NPs to enhance their efficacy in mitigating Cd toxicity. For instance, Tanveer et al. [161] used ZnO-NPs in combination with bacteria-based bio-stimulant (Biozote), which effectively improved biomass, chlorophyll synthesis, proline synthesis, membrane stability, and antioxidant as compared to the alone application of ZnO-NPs. Using ZnO-NPs and moringa (*Moringa oleifera* L.) leaf extract on linseed (*Linum usitatissimum* L.) showed a synergistic effect in improving plant growth while mitigating Cd stress. ZnO-NPs, in combination with moringa leaf extract, effectively improved antioxidant activities (APX, CAT, POD, and SOD). They decreased the MDA and H_2_O_2_ production, increasing root and shoot growth and biomass productivity under Cd stress [162]. The efficiency of Cd-tolerant PGPB, ZnO-NPs, and TiO_2_-NPs was tested on maize grown in Cd-polluted soil. The combined application of PGPB, Zn, and TiO_2_-NPs improved the defense responses, leaf water status, photosynthetic pigments, soluble sugars, proline, antioxidant activities, and nutrient uptake. The same combination also reduced the Cd bioaccumulation in roots and shoots by 40–60% [102].

A recent study investigated the impact of ZnO-NPs (0, 30, 60, 90 mg kg^−1^) and biochar (2%) in mitigating the Cd toxicity in alfalfa. The co-application of ZnO-NPs and BC decreased the Cd accumulation and improved Zn accumulation. Further, combined ZnO-NPs (90 mg kg^−1^) and BC (2%) mitigated the Cd-induced ultra-structural damages to roots and leaves and improved biomass production, antioxidant activities, and gas exchange characteristics as compared to their solo application [154]. Likewise, Bashir et al. [7] studied the impact of foliar spray of ZnO-NPs (0, 100, and 200 mg L^−1^), biochar, and farmyard manure (FYM: 0, 1, and 2%) on the performance of wheat cultivated in Cd-polluted soil. They found that the combined addition of BC, FYM (2%), and ZnO-NPs (200 mg L^−1^) significantly improved plant height, spikes, biomass, chlorophyll synthesis, and antioxidant activities and reduced the electrolyte leakage, ROS production, and Cd concentration in aerial plant parts.

The same researchers also conducted a field study to assess the impacts of ZnO nanoparticles (100 mg L^−1^) and BC (0.5%) on yield and Cd accumulation in wheat plants. The combined addition of ZnO-NPs and BC improved the growth, yield, chlorophyll synthesis, and antioxidant activities, reducing the Cd uptake in grains below the threshold levels (0.2 mg kg^−1^). In another study, an exogenous supply of Zn-NPs in combination with Si-NPs showed more promising results in improving the growth and mitigating the toxic effects of Cd in common beans [163]. These authors reported that combined Zn and Si-NPs showed promising results in decreasing MDA and EL production and improving K accumulation, antioxidant activities, spermidine (Spd) and putrescine, which helped counter Cd’s toxic effects. A very recent study finding showed that combined application of melatonin (100 μM) and ZnO-NPs (100 mg kg^−1^) improved wheat growth, chlorophyll synthesis, yield, and Zn accumulation in plant parts and decreased Cd concentration in grains [99]. Thus, combining MT and ZnO-NPs can be an important and innovative approach to minimize Cd toxicity. Recently, Anwar et al. [119] explored the role of green synthesized ZnO-NPs (25, 50, and 100 mg L^−1^) and MT (200 µM) in mitigating Cd toxicity in cauliflower *Brassica oleracea* L.) combination with MT, improved the growth traits, chlorophyll synthesis, and Zn concentration and decreased the Cd accumulation in plant parts. These authors suggested that combining ZnO-NPs and MT is a viable strategy to counteract Cd toxicity and improve Zn bio-fortification [119].

## 8. Possible Risks of Using Zinc Oxide Nanoparticles

The use of ZnO-NPs has gained significant attention from researchers. However, the safety of humans using ZnO-NPs is a key area of concern. ZnO-NPs come in contact with humans via skin, ingestion, and inhalation, and they spread throughout the body via the circulatory system. ZnO-NPs can show different biological and physiochemical characteristics; therefore, they cause unexpected risks in humans [164,165]. However, the evaluation of risks using ZnO-NPs needs more research [166,167]. ZnO-NPs are widely used in different goods, and there is also an increasing interest regarding its toxicological impacts. The consumption of emulsifiers and organic solvents during the preparation of nano-carriers can pose toxic impacts owing to their toxicological impacts [168]. There are also many concerns about using ZnO-NPs. For instance, ZnO-NPs can accumulate in food and feed; therefore, they can cause potential toxic impacts on humans [169]. Studies have reported that hazardous impacts of ZnO-NPs could be acute and chronic. Moreover, inhaling ZnO at more than 5 mg m^−3^ could pose serious risk hazards [170,171]. Thus, smart regulations are necessary to use ZnO-NPs to prevent their toxicity on humans, plants, and the environment. The strict regulation and implementation of new regulations can help to mitigate the toxicity of ZnO-NPs.

## 9. Conclusions and Future Directions

Recent industrial development has increased the Cd concentration in soil and the environment, which is a serious concern because of its toxic effects on all living organisms. Cd toxicity negatively affects plant biochemical, physiological, and molecular processes, leading to serious disruptions in growth and development. Besides this, Cd also enters the human food chain using Cd-contaminated crops, jeopardizing health issues. ZnO-NPs have proven to be key players in mitigating Cd toxicity and its accumulation in plants. Applying ZnO-NPs has shown an appreciable ability to improve photosynthesis, plant water relation, membrane stability, chlorophyll synthesis, and nutrient uptake, leading to a marked increase in plant performance. Further, ZnO-NPs also improve gene expression, antioxidant activities, osmolytes, and secondary metabolite synthesis and reduce Cd accumulation, which help the plants to withstand Cd toxicity and ensure better plant growth. Globally, efforts are being made to clarify the role of ZnO-NPs in mitigating Cd toxicity in plants; nevertheless, many unanswered questions exist.

- Zinc plays a crucial role in seed germination; however, the role of ZnO-NPs in seed germination in Cd-polluted soil has yet to be understood. Therefore, it will be interesting to investigate how ZnO-NPs can improve seed germination and subsequent seedling growth in Cd-polluted environments.
- In the literature, ZnO-NPs have shown promising results in improving nutrient uptake. However, no study has investigated the mechanism by which ZnO-NPs can improve nutrient uptake in Cd-polluted soil. Therefore, exploring how ZnO-NPs can improve nutrient uptake in Cd-polluted soil is essential.
- The anastomotic changes induced by ZnO-NPs in response to Cd toxicity must be explored to better understand the role of ZnO-NPs under Cd stress.
- Phyto-hormones play an essential role in reducing Cd stress; however, more information is needed in the literature regarding the effect of ZnO-NPs on phyto-hormone synthesis. Thus, exploring the complex relationship between ZnO-NPs and phyto-hormones is mandatory to ensure Cd tolerance in plants while using ZnO-NPs.
- It would also be interesting to explore the ZnO-NPs signaling mechanism at cell, tissue, and organ levels to counter Cd toxicity.
- Recently, the combined use of ZnO-NPs and different microbes, hormones, osmolytes, and organic amendments showed promising results in countering Cd toxicity. Therefore, further research is required to investigate the mechanism of the co-application of ZnO-NPs and other amendments to alleviate Cd stress.
- On a field scale, soils are mostly contaminated with different toxic metals; therefore, new research must explore the effect of ZnO-NPs on soils contaminated by multiple heavy metals.
- Recently, biosynthesized ZnO-NPs showed promising results against Cd toxicity. However, more research is needed to explore how green-synthesized ZnO-NPs can mitigate Cd toxicity.
- The effect of ZnO-NPs on the expression of Cd transport genes in roots must also explored to better understand how ZnO-NPs affect Cd uptake and transport. Investigations are also needed to study the influence of climate change on the efficacy of ZnO-NPs to immobilize Cd. The fluctuations in rainfall, temperature and other climate conditions can change the fate of Cd in soil-plant system.
- The future studies should also explore the impacts of ZnO-NPs on soil microbes and the overall health of an ecosystem. An understanding of the consequences of using ZnO-NPs to remediate Cd-polluted soils is essential to develop environmental approaches. The effect of ZnO-NPs is mostly investigated in controlled conditions, and it is highly recommended that the role of ZnO-NPs be understood against Cd toxicity in field conditions. It is also essential to optimize application rates of ZnO-NPs by soil, seed priming, and foliar sprays while considering the soil and climatic conditions.
- In the literature, there is no information available about the cost of ZnO-NPs to remediate Cd-contaminated soils. Therefore, in the future, authors must add the cost of ZnO-NPs in remediating Cd-polluted soils for a promising future of ZnO-NPs.

## Figures and Tables

**Table 1:** Effect of cadmium stress on growth, physiological functioning, nutrient homeostasis, osmolyte synthesis, and antioxidant activities of different plant species.

Plant Species	Cd Concentrations	Route of Administration	Growth Media	Major Effects	References
*Helianthus annuus*	0, 0.15, and 0.25 mg kg^−1^	Soil application	Pot	Cd toxicity reduced seed germination, seedling growth and biomass, chlorophyll, carotenoid, phenolic, and flavonoid while Cd stress augmented the malondialdehyde (MDA: (45.24–53.06%), H_2_O_2_ (−11.07% and 5.86%), and soluble sugars (28.05–50.34%) as compared to no Cd stress.	[79]
*Lactuca sativa*	10 and 20 mg kg^−1^	Soil application	Pot	Cd stress reduced chlorophyll content, damaged electron transport, reduced energy transfer, and inhibited photosynthesis.	[80]
*Zea mays*	0, 1, 3, 5, and 7 mg L^−1^	Soil application	Pot	Cd stress decreased aboveground and belowground biomass, leaf water content, and increased, superoxide dismutase (SOD), peroxidase (POD), and catalase (CAT) and membrane lipid peroxidation.	[81]
*Oryza sativa*	0, 2, and 10 mg kg^−1^	Soil application	Pot	Cd stress significantly damaged rice roots and leaves, inhibited plant growth, and altered metabolic profiles by down-regulating glycerol–phospholipid metabolism in stem and leaf tissues.	[82]
*Elymus dahucirus*	0, 40, 90, 140, and 190 μM L^−1^	Soil application	Pot	Cd stress decreased plant growth and increased proline synthesis, antioxidant enzyme burst, and MDA production.	[83]
*Sesuvium portulacastrum*	0–600 µM	Nutrient medium	Pot	Cd stress led to a decline in roots, leaf tissues, stems, and chlorophyll content and an increase in SOD and POD activity. Moreover, Cd stress also disturbed the structure of roots, stems, and leaves and deformed the chloroplasts, and mitochondria.	[84]
*Zea mays*	25 mg kg^−1^	Soil application	Pot	Cd treatment significantly reduced morphological attributes, photosynthetic pigments, and cell membrane stability and increased MDA, electrolyte leakage (EL), and hydrogen peroxide (H_2_O_2_) levels.	[85]
*Tradescantia pallida*	100 and 200 μM L^−1^	Hoagland solution	Pot	Cd toxicity decreased glutathione content, photosynthetic capacity, antioxidant enzyme activities, and intercellular CO_2_ concentration and increased proline, soluble proteins, soluble sugars, and MDA concentration.	[86]
*Glycine max*	0 and 50 µM	Soil application	Pot	Cd stress significantly reduced shoot dry weight, chlorophyll content, and total glutathione content in soybean leaves, while increasing electrolyte leakage increased MDA and H_2_O_2_ production.	[87]
*Brassica oleracea*	25 μM L^−1^	Soil application	Pot	Cd stress decreased quantum yield of PS-II photochemical efficiency of PS-II photochemical quenching coefficient 78.5%, 68.51%, and 59.55%. Further, Cd stress also decreased growth, photosynthetic pigment, and gas exchange characteristics, disrupted chloroplast structure, and caused oxidative damage and increased accumulation of O_2_−, H_2_O_2_, MDA, electrolyte leakage, and proline.	[88]
*Ipomoea batatas*	0.27 mg kg^−1^	Soil application	Pot	Cd toxicity significantly inhibited vine length, leaf area, stem diameter, individual plant dry matter weight, branch number, chlorophyll synthesis, and Rubisco activity and increased antioxidant activities.	[89]
*Triticum aestivum*	0, 2.5, 5, 10 mg kg^−1^	Soil application	field	Cd toxicity reduced spike length and root and shoot length by 26%, 59%, and 46%. Under Cd stress, chlorophyll contents were also decreased, while EL, MDA levels, H_2_O_2_ concentration, and Cd uptake and accumulation increased in plant parts and grains.	[90]
*Capsicum annuum*	0, 3, 4, and 5 mg kg^−1^	Soil application	Pot	Cd toxicity the plant growth, carotenoid (−197.39%), flavonoids (−37.63%), POD (−31.18%), CAT (−16.39%), SOD (−25.98%) activity, and increased translocation of Cd to plant parts.	[91]
*Pisum sativum*	0, 50, and 100 μM	Soil application	Pot	Cd toxicity decreased plant growth, chlorophyll content, osmoprotectants, and anthocyanin content and increased MDA, H_2_O_2_, enzymatic antioxidant activities, phenolic, flavonoid, and proline synthesis.	[92]
*Solanum lycopersicum*	0, 12, and 25 μM	Soil application	Pot	Cd stress negatively impacted chlorophyll content, chlorophyll fluorescence, photosynthesis efficiency, growth traits, and increased Cd accumulation in plant parts.	[93]
*Polygonatum cyrtonema*	0.96 and 3.86 mg kg^−1^	Soil application	Pot	Cd stress led to a significant decrease in biomass due to oxidative damage. However, Cd toxicity also decreased choline, indole acetic acid (IAA), and fatty acid metabolites. Further, the metabolism of caffeine, glutamine, arginine, and purine was up-regulated in response to Cd stress.	[94]
*Gossypium herbaceum*	0, 200, and 400 μM	Nutrient solution	Pot	Cd toxicity reduced the root and leaf biomass and results in production of slender and dehydrated plants. Further Cd stress also decreased glycine, cysteine, and glutamic acid and increased axcorbate peroxidase (APX), catalase (CAT), POD, SOD, and GR activities, root and shoot Cd concentration, and copper (Cu), magnesium (Mg), potassium (K), and manganese (Mn) concentration in roots and shoots.	[95]
*Zea mays*	15 and 30 mg kg^−1^	Nutrient solution	Pot	Cd toxicity resulted in minimum shoot length, shoot weight, chlorophyll, carotenoid, soluble proteins, free amino acids, APX, CAT, POD, and SOD activities and root Cd concentration as compared to control.	[96]
*Triticum aestivum*	0.5 mM	Soil application	Pot	Cd toxicity significantly increased ROS, MDA, and EL and decreased growth, yield, oxidized glutathione contents, chlorophyll, carotenoid, Ca, and K uptake.	[97]
*Capsicum annuum*	0, 2, and 4 mM	Soil application	Pot	Cd markedly reduced the plant biomass (18 and 40%), RWC (11 and 24%), chlorophyll contents (16 and 31%), and increased EL (49 and 129%) and MDA contents (68 and 104%) and root and fruit Cd concentration.	[98]

## Data Availability

Not applicable.
